# Experimental Confirmation of the Role of the α1–α2 Hairpin PVY VPg in eIF4E Interaction

**DOI:** 10.3390/ijms27188187

**Published:** 2026-09-15

**Authors:** Victoria Kolesnikova, Alisa Mikhailina, Natalia Lekontseva, Oleg Nikonov, Stanislav Nikonov, Vladimir Andreytsev, Eugene Maksimov, Do Tien Phat, Ngoc Thu Le, Ekaterina Nikonova

**Affiliations:** 1 Institute of Protein Research, Russian Academy of Sciences, 142290 Pushchino, Russia; kolesnikovavicto@yandex.ru (V.K.); vvlad.andr@gmail.com (V.A.); 2 Faculty of Biology, Moscow State University, Leninskye Gory 1, Build. 12, 119234 Moscow, Russia; 3 Institute of Biology, Vietnam Academy of Science and Technology, Hanoi 100000, Vietnam

**Keywords:** VPg, eIF4E, protein engineering, SPR, protein purification

## Abstract

In recent years, the eIF4E protein family has been actively studied as one of the major susceptibility factors for Solanaceae plants in relation to potyvirus infections. This makes eIF4E an attractive target for genome editing to generate resistant varieties. The viral protein VPg interacts with eIF4E family proteins. Measuring the in vitro affinity of VPg for different eIF4E isoforms has traditionally been difficult. The main reason for this is the high aggregation propensity of VPg. To overcome this, we used protein engineering to generate a chimeric construct based on a fluorescent protein. This approach greatly facilitated the measurement of affinity between two proteins using surface plasmon resonance (SPR). Our results confirm that the α1–α2 hairpin of PVY VPg is involved in complex formation with eIF4E. These findings provide new insights into the molecular mechanism of this protein–protein interaction.

## 1. Introduction

Potato virus Y (PVY) is a widespread virus that affects a broad range of agricultural crops [1,2]. The most significant damage affects economically important agricultural crops from the Solanaceae family, such as tobacco, potato, pepper, tomato, and others. PVY possesses a positive-sense single-stranded RNA genome with the viral genome-linked protein (VPg) covalently attached to its 5′ terminus [3,4].

Susceptibility to potyviruses in Solanaceae plants has been linked to genes encoding the eIF4E protein family [5,6,7,8,9]. As eIF4E is required for the viral life cycle, it is a key host susceptibility factor for PVY in plants [10,11]. eIF4E is a cap-binding protein that plays a key role in translation initiation [12,13,14]. Our research is focused on the complex between eIF4E and PVY VPg, which is central to virus susceptibility.

Despite the availability of structural data for free VPg and eIF4E homologs from various species (including mouse, human, and yeast), no experimental structure of the eIF4E∙VPg complex has been reported to date. Structural studies of eIF4E homologs reveal an extended disordered N-terminal region that hinders crystallization of the full-length protein. NMR data on PVY VPg confirm that this protein also contains disordered and flexible regions (PDB: 1AP8) [15].

Our group has previously proposed a model of the eIF4E∙VPg complex and identified a putative region involved in binding [16,17]. According to our model, for complex formation, all amino acid residues from PVY VPg that mediate the interaction are concentrated within the α1–α2 hairpin (residues 96–124). That hairpin of PVY VPg interacts with eIF4E in the cap-binding pocket. This model is also corroborated by modeling of other potyviral VPgs, which reveals a conserved interaction module [18]. Validation of this model requires either an experimental structure of the complex and/or functional assays. If the isolated PVY VPg region (the α1–α2 hairpin), which is involved in interaction with eIF4E, interacts with eIF4E under in vitro conditions, then this will be a strong argument in favor of the model.

In this study, we applied molecular modeling methods to create theoretical models of tomato eIF4E, PVY VPg, and the chimeric protein mCherry-VPg. We also used the molecular dynamics method to evaluate the stability of the obtained models. Based on these results, we obtained recombinant tomato eIF4E1 and PVY VPg to measure their affinity, and then applied a protein engineering strategy to map the VPg–eIF4E interaction interface and to optimize the assay for measuring the binding affinity.

## 2. Results and Discussion

### 2.1. Molecular Modeling of SleIF4E

The PDB contains extensive structural data on eIF4E homologs across many species. Nevertheless, structural information on plant eIF4E family proteins remains scarce. No experimental structures have yet been reported for eIF4E proteins from Solanaceae plants. Using the abundant structural data on eIF4E homologs, we built a high-confidence theoretical model of eIF4E of *Solanum lycopersicum* (SleIF4E) with AlphaFold3. The family of SleIF4E comprises several isoforms: eIF4E1, eIF4E2, eIF(iso)4E, and nCBP. All the experiments described here were carried out on the eIF4E1 isoform. The optimized protocol can later be extended to the remaining isoforms of this family.

**Figure 1:**
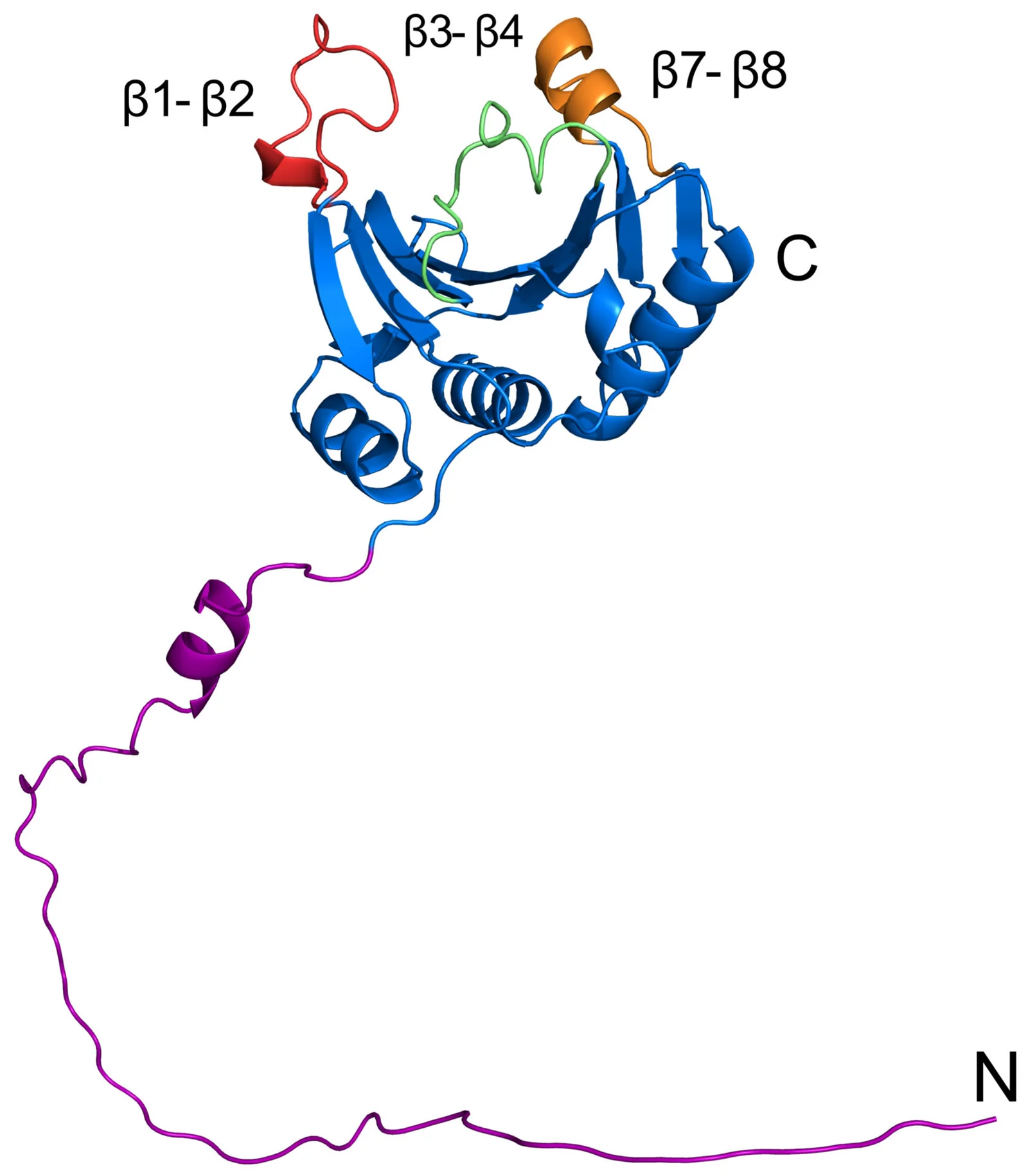
Theoretical model of SleIF4E1. The globular domain and unstructured N-terminal region are shown in marine and purple, respectively. The cap-binding pocket is formed by the β1–β2 (red), β3–β4 (green), and β7–β8 (orange) loops.

The theoretical model of SleIF4E1 was built with the help of Alphafold3. The model comprises a globular domain and an unstructured N-terminal fragment (residues 1–53, Figure 1). The globular part of the protein includes the major functional regions of eIF4E: the cap-binding pocket and the eIF4G interaction site. This fold is conserved among eIF4E homologs with known experimental structures.

The resulting model was refined and subsequently used as the starting structure for a 400 ns molecular dynamics trajectory. The molecular dynamics simulations showed that the unstructured N-terminal region is highly mobile. The NMR structure of an eIF4E homolog from *Saccharomyces cerevisiae* (PDB: 1AP8) confirms the high flexibility of this region. This region in homologous eIF4E for crystallization is usually removed. Removing this region is unlikely to perturb the cap-binding pocket, which is formed by three loops and a β-sheet. This is supported by experimentally determined structures of homologous truncated pea eIF4E in complex with a cap analogue (PDB: 2WMC), mouse eIF4E in complex with a cap analogue (5J5O, 5J5Y, 5M7W). In addition, the absence of the N-terminal region does not affect the ability of eIF4E homologues from other organisms to interact with eIF4G and 4E-BP, which is supported by experimentally determined structures of homologous truncated eIF4E in complex with 4E-BP (5ABV, 5ABY) and 4E-binding motif eIF4G (6FC0). The resulting truncated construct was named SleIF4E1-Δ1-54. This compact variant can therefore be used in future crystallization trials, as its functional activity is not expected to be compromised.

### 2.2. Purification of SleIF4E1-Δ1-54

The nucleotide sequence encoding SleIF4E1-Δ1-54 was subcloned into the pET22b expression vector. The protein was subsequently overexpressed, isolated, and purified (see Section 3), and the resulting sample was used in all further experiments.

### 2.3. Purification of PVY VPg

The PVY VPg coding sequence was cloned into the pET22b expression vector, and the conditions for protein expression and purification were optimized as described in Section 3.

Purification of full-length PVY VPg revealed that this protein is highly aggregation-prone. Size-exclusion chromatography showed that the majority of the protein elutes in inactive oligomeric forms, while the active monomer represents only ~10% of the total. The fractions containing the oligomeric and monomeric forms were concentrated separately. As a consequence, even upon high-level overexpression, only limited quantities of protein suitable for functional studies are recovered after purification. Furthermore, we observed complications with immobilization of PVY VPg on SPR sensor chips. Given the aggregation tendency of full-length VPg and the difficulties associated with its immobilization on SPR chips, we decided to generate a chimeric construct that would circumvent these limitations and make the assay more cost-effective.

### 2.4. Creation of a Theoretical Model of Chimeric Protein

We used the stable fluorescent protein, mCherry, as a molecular scaffold to create the chimeric construct. The structure of mCherry has been experimentally determined, providing a reliable framework for the chimeric design (PDB 2H5Q). According to our previously created model of the eIF4E∙VPg complex, PVY VPg binds to eIF4E within the cap-binding pocket [17]. For complex formation all amino acid residues from PVY VPg that mediate the interaction are concentrated within the α1–α2 hairpin. According to our model, R104 in the α1–α2 hairpin of PVY VPg (numbered according to accession KC005979) and the E115 residue in the β3–β4 loop of eIF4E (numbered according to accession MT828873) are the key determinants of this interaction.

**Figure 2:**
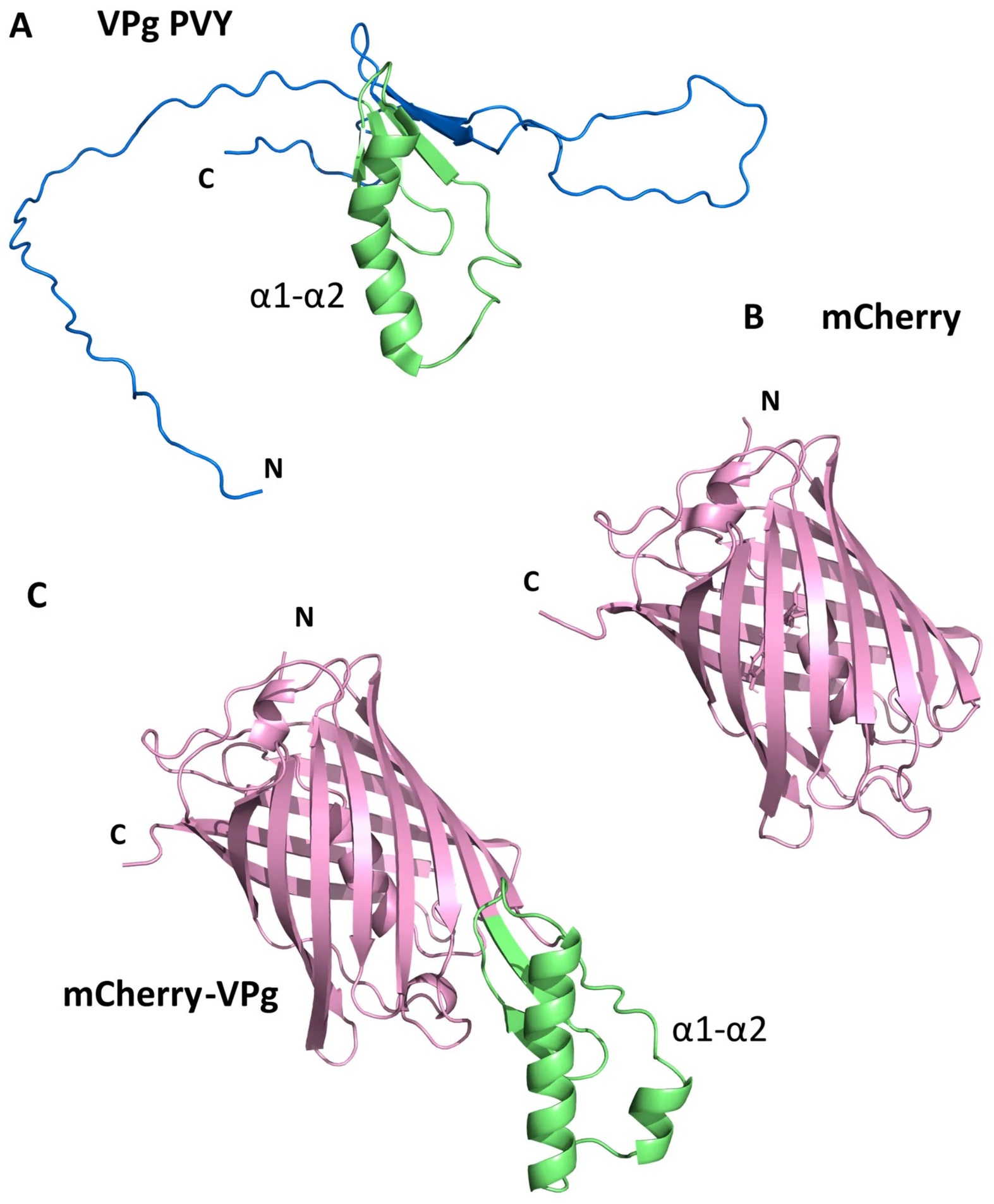
Models of individual proteins and the chimeric construct: (**A**) NMR structure of PVY VPg (PDB 6NFW). PVY VPg is colored in marine. The region corresponding to residues 74–128 of PVY VPg is highlighted in green. (**B**) Crystal structure of mCherry (PDB 2H5Q). mCherry is colored pink. (**C**) Theoretical model of the mCherry-VPg chimera, with mCherry shown in pink and residues 74–128 of PVY VPg highlighted in green.

The sequence encoding the α1–α2 hairpin region of PVY VPg (residues 74–128) was inserted between V35 and N36 of mCherry (UniProtKB accession D1MPT3 numbering). This region corresponds to the β1–β2 loop of mCherry. We then generated a model using AlphaFold3, which was refined and used as the starting model for a 400 ns molecular dynamics simulation (Figure 2). In this chimeric version, the α1–α2 hairpin of PVY VPg proved to be stable (Figure S1).

### 2.5. Purification of Chimeric Protein mCherry-VPg

The chimeric construct was assembled using the IVA cloning method (see Section 3). This approach enables insertion of diverse nucleotide sequences at any position, without restriction site dependence. This greatly simplifies and accelerates genetic construct assembly. The protein was overexpressed, extracted, and purified (see Section 3).

The chimeric protein was isolated from inclusion bodies and efficiently refolded. The reason for the efficient renaturation of this protein lies in its β-barrel scaffold, which is a defining feature of fluorescent proteins. This architecture confers exceptional thermodynamic stability and enables rapid refolding even after denaturation. Furthermore, two-step purification from inclusion bodies yielded protein of high purity, which was used in all the subsequent experiments.

### 2.6. SPR Analyses

**Figure 3:**
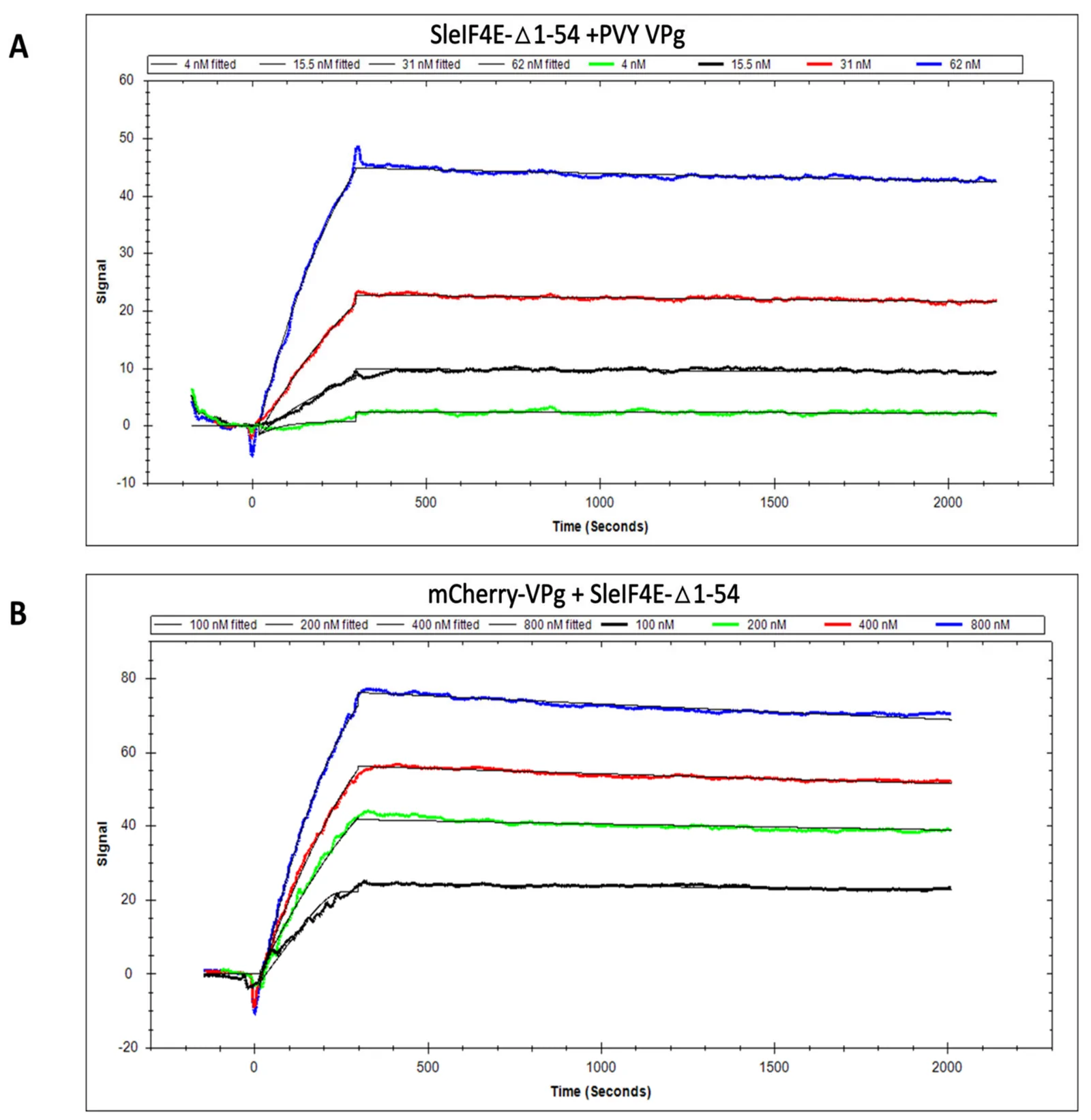
Sensorgrams of interaction of SleIF4E1-Δ1-54 and PVY VPg (**A**) and mCherry-VPg (**B**). (**A**)—thick colored lines represent experimental data at different analyte (PVY VPg) concentrations: blue—62 nM; red—31 nM; green—4 nM; gray—15.5 nM; (**B**)—thick colored lines represent experimental data at different analyte (SleIF4E1-Δ1-54) concentrations: blue, 600 nM; red, 400 nM; green, 200 nM; gray, 100 nM; Thin black lines correspond to the global fitting curves. In several traces, the experimental and fitted curves are superimposed, indicating an excellent correlation between the experimental data and the binding model.

To determine the affinity between SleIF4E1-Δ1-54 and full-length PVY VPg in vitro, surface plasmon resonance (SPR) was used. Initially, immobilization of PVY VPg on the chip did not allow us to detect binding. This was most likely because the binding site for SleIF4E1-Δ1-54 was occluded upon immobilization of PVY VPg. To analyze the interaction, SleIF4E1-Δ1-54 was immobilized on the sensor chip, and PVY VPg was used as the analyte (Figure 3). Only the monomeric form of PVY VPg showed the ability to interact with SleIF4E1-Δ1-54. The oligomeric form of PVY VPg does not interact with the immobilized ligand. This experimental setup allowed us to obtain data and calculate the dissociation constant (KD) of the complex (Table 1). However, this approach requires a new chip for each protein to determine kinetic parameters, which is a significant drawback.

A similar experiment was performed with the chimeric mCherry-VPg construct. This approach pursued three goals. The first was to assess the ability of mCherry-VPg to interact with SleIF4E1-Δ1-54. The second was to verify the functional role of the α1–α2 hairpin of PVY VPg in complex formation with eIF4E. The third was to assess the suitability of this chimeric construct as an immobilized ligand on the SPR chip.

So, in the next experiments the chimeric protein mCherry-VPg was used as the immobilized ligand on the chip, while SleIF4E1-Δ1-54 was used as the analyte (Figure 3). The data are presented in Table 1. Immobilization of mCherry-VPg on the chip allows the same chip to be used for sequential measurement of kinetic parameters with different analytes. This configuration spatially separates the interaction site on the α1–α2 hairpin of PVY VPg from the chip surface. Our chimeric construct thus offers a simplified, more cost-effective, and faster approach for analyzing PVY VPg interactions with various eIF4E isoforms.

To rule out nonspecific binding of mCherry-VPg to other proteins, BSA was used as a negative control and applied as the analyte (Table 1). To exclude any contribution of the mCherry moiety to the interaction between the chimeric mCherry-VPg construct and SleIF4E1-Δ1-54, we tested whether the isolated mCherry protein alone can bind SleIF4E1-Δ1-54. This experiment confirmed the absence of such an interaction (Table 1).

Our data indicate that SleIF4E1-Δ1-54interacts with both PVY VPg and the chimeric mCherry-VPg protein with the same affinity (Table 1). Moreover, the obtained dissociation constant indicates the formation of a stable, specific complex. This confirms the role of the α1–α2 hairpin of PVY VPg in complex formation with SleIF4E1-△1-54. Furthermore, the obtained data is in good agreement with our previously proposed theoretical model.

Our chimeric construct can be used to screen the affinity of PVY VPg for different eIF4E isoforms from different plant species, enabling the identification of the most suitable targets for further study and genome editing.

## 3. Materials and Methods

### 3.1. Molecular Modeling and Molecular Dynamics

Models of SleIF4E1-Δ1-54 were created using amino acid sequences from the Phytozome (CDS of Gene ID: Solyc03g005870) and the AlphaFold3 algorithm [19]. Of the five models generated by the algorithm, model 0 (with the highest pLDDT value) was selected for further refinement.

Molecular dynamics (MD) simulations were performed using the GROMACS package (2024.6). CHARMM36 (2022 release) was used as the force field. The spatial configuration of the studied systems (protein and protein–protein complex) was placed at the center of a computational cell shaped like a rhombic dodecahedron. The cell size was chosen such that the minimum distance from any atom of the molecule to the cell boundary was 1.5 nm. The cell was filled with water molecules using the TIP3P model. To achieve electroneutrality of the system, Na^+^ and Cl^−^ counterions were added at a concentration sufficient to compensate for the net charge of the macromolecules.

Before the main simulation stage, energy minimization was performed using the steepest descent method. The system was then equilibrated in two stages. First, relaxation was performed in the NVT ensemble to bring the system to the target temperature of 310 K. Next, equilibration was performed in the NPT ensemble (constant particle number, pressure, and temperature) at a pressure of 1 bar and a temperature of 310 K. A v-rescale thermostat and a C-rescale barostat were used for both equilibration stages. The final production stage of the MD simulation was performed in the NPT ensemble using the V-rescale thermostat and the Parrinello-Rahman barostat for 400 ns with an integration step of 2 fs. Trajectory recordings were performed at 10 ps intervals.

### 3.2. Data Analysis and Visualization

Superposition and comparative analysis of experimental and theoretical models were performed using the COOT and PyMOL software packages (PyMOL Version 2.6.2, OPEN-SOURCE). Analysis and visualization of the MD trajectories were carried out using VMD software, version 2.0.0.

### 3.3. Plasmid Construction

#### 3.3.1. Plasmid pET22-SleIF4E1-△1-54

Total RNA was isolated from the leaves of *Solanum lycopersicum* plants using TRIzol Reagent (Thermo Scientific) according to the manual instructions. cDNA was obtained using the RevertAid First cDNA Synthesis Kit (Thermo Scientific) according to the manual instructions. Primers for amplification of the target fragment, which encodes eIF4E1, were designed based on a sequence from the Phytozome (Gene ID: Solyc03g005870). The sequence of eIF4E1 of *Solanum lycopersicum* was cloned into the pJET1.2 vector with the help of the CloneJET PCR Cloning Kit (Thermo Scientific, U.S.A.) and was sequenced by Sanger sequencing.

**Figure 4:**
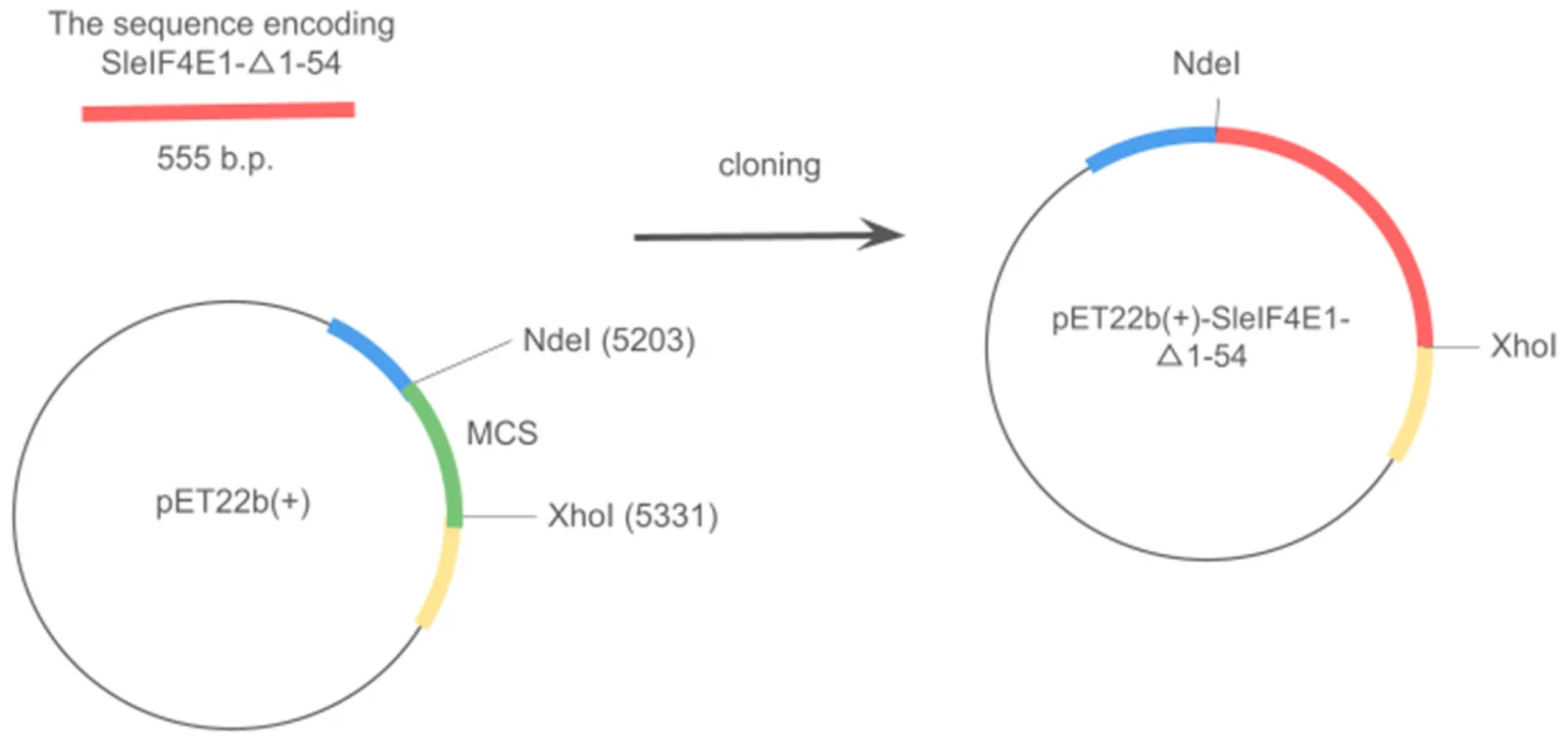
A schematic representation of the process of creating an expression vector pET22b(+)-SleIF4E1-Δ1-54. The T7 promoter region is marked in blue. The T7 terminator region is marked in yellow. The multiple cloning site (MCS) is marked in green.

Subsequently, the coding sequence corresponding to the proposed globular domain of *Solanum lycopersicum* eIF4E1 (SleIF4E1-△1-54), spanning nucleotide positions 160 to 693 of the open reading frame, was amplified by PCR using flanking primers (1F 5′-ACCAGTCACATATGGTGAAGCATCCATTGGAGCATTC-3′ and 1R 5′-ACTGACTCTCGAGTACGGTGTAACGATTCTTGGCATTTCTG-3′) that introduced NdeI and XhoI restriction sites at the 5′ and 3′ ends, respectively. The primers also contained additional 5′ overhangs to ensure efficient enzymatic hydrolysis. PCR was performed in a total volume of 50 µL containing 10 ng of plasmid DNA, 0.5 µM of each primer, 200 µM dNTPs, 1× Phusion HF buffer, 1U of Phusion High-Fidelity DNA Polymerase (Thermo Fisher Scientific), and nuclease-free water. The thermal cycling conditions were as follows: initial denaturation at 98 °C for 3 min; 30 cycles of denaturation at 98 °C for 30 s, annealing at 56 °C for 30 s, and extension at 72 °C for 60 s; followed by a final extension at 72 °C for 10 min. The expected amplicon size was 555 bp. The resulting PCR products were resolved by electrophoresis on a 1.5% (*w*/*v*) agarose gel in 1× TAE buffer, stained with SYBR Green, and visualized under UV light. The amplicon of the correct size was purified using the QIAquick PCR Purification Kit (Qiagen). This amplicon and the pET22b expression vector (Novagen) were separately digested with XhoI and NdeI (Thermo Fisher Scientific). The restriction digestion mixture contained 1× buffer O, 300 ng of DNA (vector or insert), 10 U of each restriction enzyme, 0.1 mg/mL BSA, and nuclease-free water in a final volume of 50 µL. The mixture was incubated at 37 °C for 2 h, then at 50 °C for 1 h, followed by heat inactivation at 60 °C for 10 min. The digested products were purified using the QIAquick PCR Purification Kit (Qiagen) according to the manufacturer’s instructions. The purified, linearized vector and the digested insert were ligated using T4 DNA ligase (Thermo Fisher Scientific, Massachusetts, WA, USA) at a molar ratio of 1:7 (vector:insert). The ligation reaction was incubated at 16 °C for 16 h. The ligase mixture was used directly to transform chemically competent *E. coli* XL1-Blue cells. The positive clones were selected on LB agar plates supplemented with 100 µg/mL ampicillin and verified by colony PCR. The resulting construct, encoding SleIF4E1-Δ1-54with a C-terminal His_6_-tag, was named pET22b(+)-SleIF4E1-Δ1-54 (Figure 4).

#### 3.3.2. Plasmid pET22-VPg PVY

**Figure 5:**
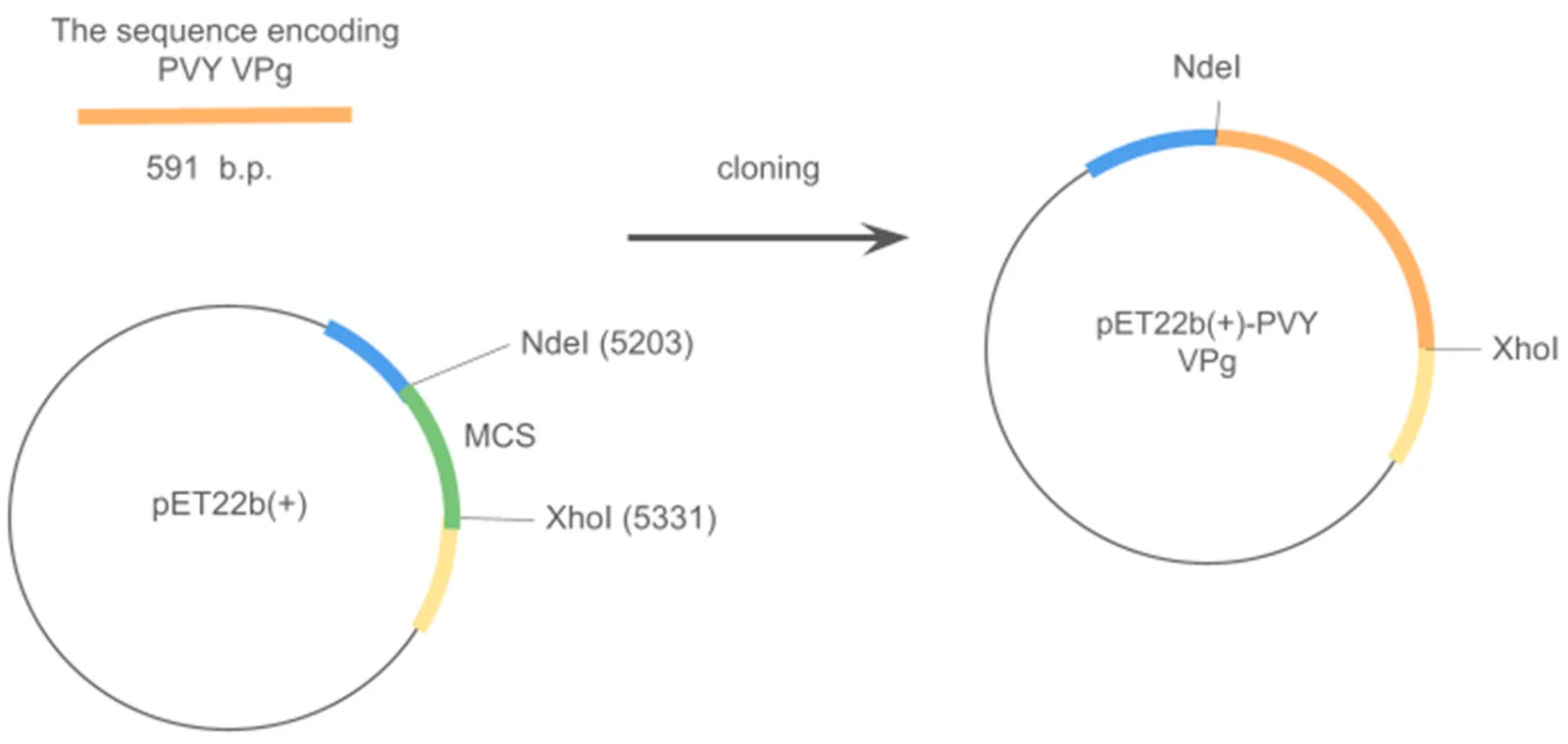
A schematic representation of the process of creating an expression vector pET22b(+)-PVY VPg. The T7 promoter region is marked in blue. The T7 terminator region is marked in yellow. The multiple cloning site (MCS) is marked in green.

Total RNA was isolated from the leaves of *Solanum tuberosum* L., of the Zhukovsky Ranniy cultivar, using the ExtractRNA Reagent kit (Evrogen, Moscow, Russia) according to the manual instructions. cDNA was obtained using ProtoScript^®^ II Reverse Transcriptase (NEB, Milton Park, Oxfordshire, UK) according to the manual instructions. To generate the expression construct for the PVY VPg, the corresponding cDNA fragment was amplified by PCR using flanking primers (2F 5′-ACCAGTCACATATGGGGAAAAATAAATCCAAAAGAATTCAAGCC-3′, 2R 5′-ACTGACTCTCGAGTTCATGCTCCACTTCCTGTTTTGGAATGTC-3′) that introduced NdeI and XhoI restriction sites at the 5′ and 3′ ends, respectively. The amplification fragment VPg PVY was made as in Section 3.3.1. The PCR cycling conditions were as follows: initial denaturation at 95 °C for 5 min; 30 cycles of denaturation at 95 °C for 40 s, annealing at 53 °C for 40 s, and extension at 72 °C for 1 min; followed by a final extension at 72 °C for 10 min. The expected amplicon size was 591. The resulting PCR products were resolved by electrophoresis on a 1.5% (*w*/*v*) agarose gel in a 1× TAE buffer, stained with SYBR Green, and visualized under UV light. The amplicon was purified using a QIAquick PCR Purification Kit (Qiagen, Germany). The purified fragment was then cloned into the pET22b expression vector (Novagen, Darmstadt, Germany) as in Section 3.3.1. The resulting construct, encoding PVY VPg with a C-terminal His_6_-tag, was named pET22b(+)-PVY VPg (Figure 5).

#### 3.3.3. Plasmid pET24-mCherry-VPg

**Figure 6:**
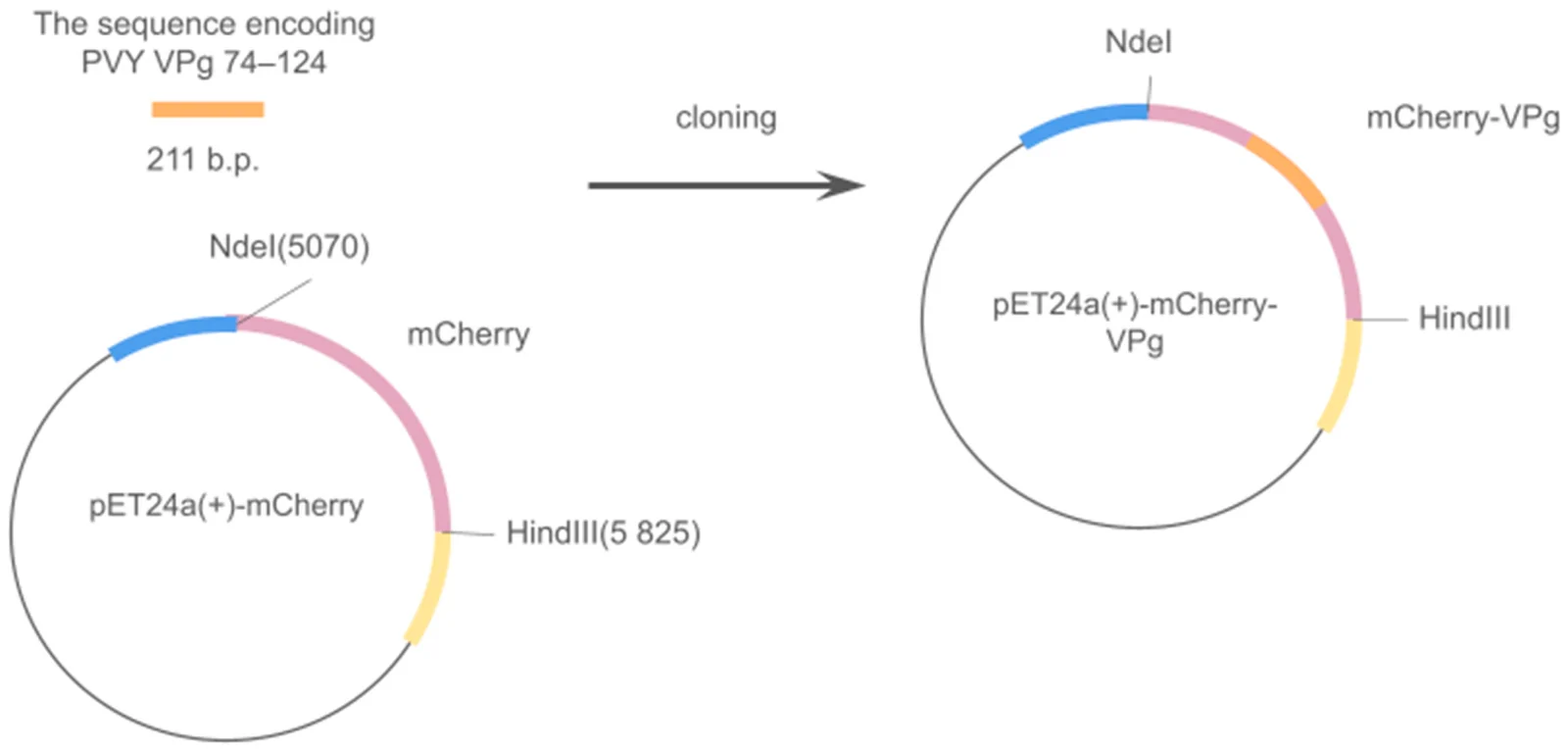
A schematic representation of the process of creating an expression vector pET24a(+)-mCherry-VPg. The T7 promoter region is marked in blue. The T7 terminator region is marked in yellow.

The genetic construct for expression of the chimeric protein was generated via in vivo assembly or IVA [20]. The mCherry gene was amplified from plasmid pET24a(+)-mCherry using primers 3F (5′-AACGGCCACGAGTTCGAGATCGAGGGCGAGG-3′) and 3R (5′-CACGGAGCCCTCCATGTGCACCTTGAAGC-3′). PCR was performed in a total volume of 50 µL containing 5 ng of plasmid DNA, 0.3 µM of each primer, 0.2 mM dNTPs, 1× buffer, 1U of KOD HS DNA Polymerase (Novagen), and nuclease-free water. The thermal cycling conditions were as follows: initial denaturation at 98 °C for 5 min; 18 cycles of denaturation at 98 °C for 40 s, annealing at 61 °C for 40 s, and extension at 72 °C for 8 min; followed by a final extension at 72 °C for 10 min. The expected amplicon size was 6083 b.p. The resulting PCR products were resolved by electrophoresis on a 1% (*w*/*v*) agarose gel in a 1× TAE buffer, stained with SYBR Green, and visualized under UV light. The fragment of VPg encoding the 74–128 amino acid region was amplified from plasmid pET22b(+)-VPg-PVY using primers F (5′- CCTCGCCCTCGATCTCGAACTCGTGGCCGTTGAAGTAAGCATGAATGGTTGTGTTGCTGCCCAATGC-3′) and R (5′- GCTTCAAGGTGCACATGGAGGGCTCCGTGATTCAGTTCGTTGATCCGCTCACTGGAGCTCAAATTG-3′). PCR was performed in a total volume of 50 µL containing 5 ng of plasmid DNA, 0.3 µM of each primer, 0.2 mM dNTPs, 1× buffer, 0.02 U of KOD HS DNA Polymerase (Novagen), and nuclease-free water. The thermal cycling conditions were as follows: initial denaturation at 98 °C for 5 min; 20 cycles of denaturation at 98 °C for 40 s, annealing at 61 °C for 40 s, and extension at 72 °C for 1 min; followed by a final extension at 72 °C for 10 min. The expected amplicon size was 211 b.p. The resulting PCR products were resolved by electrophoresis on a 1.5% (*w*/*v*) agarose gel in a 1× TAE buffer, stained with SYBR Green, and visualized under UV light. The amplicons were treated with MalI and used directly to transform chemically competent *E. coli* XL1-Blue cells at a 1:10 molar ratio of vector to insert. Finally, this genetic construct is named pET24a(+)-mCherry-VPg (Figure 6).

### 3.4. Expression and Purification of Recombinant Proteins

#### 3.4.1. Expression and Purification of Recombinant eIF4E1 of *Solanum lycopersicum*

Cells of *E. coli* strain BL21(DE3)pRARE (Novagen, Germany) were transformed with plasmid pET22b(+)-SleIF4E1-Δ1-54 and grown on selective agar medium supplemented with 100 µg/mL ampicillin for 16 h at +37 °C. A single colony from the plate was transferred into 10 mL of selective LB medium and incubated overnight at +37 °C with shaking at 180 rpm. The overnight culture was subcultured into a preparative volume of selective medium (100 µg/mL ampicillin) at a 1:50 ratio. Protein expression was induced by adding 0.3 mM IPTG (final concentration) when the culture OD590 reached ≈0.6–0.8. Cells were incubated for 3 h at +37 °C. The expression level was checked using SDS-PAGE. Cells were harvested by centrifugation.

To obtain SleIF4E1-Δ1-54, harvested cells were resuspended in buffer R1 (10 mM Tris-HCl pH 7.5, 250 mM NaCl, 10% glycerol) supplemented with DNAse and PMSF. The cell suspension was disrupted by sonication in an ice-water bath. After that, the suspension was centrifuged at 10,000 rpm (13751 rcf) for 30 min at +4 °C. The target proteins were in the insoluble fraction. The debris was resuspended in buffer R2 (10 mM Tris-HCl pH 7.5, 250 mM NaCl, 2M urea, 5 mM imidazole), then centrifuged at 10,000 rpm (13751 rcf) for 30 min at +4 °C. The resulting pellet was then resuspended in buffer R3 (10 mM Tris-HCl pH 7.5, 250 mM NaCl, 6M urea, 5 mM imidazole), and centrifuged again under the same conditions. The supernatant obtained after the final centrifugation contained the target protein SleIF4E1-Δ1-54. This supernatant containing denatured proteins was applied to the column with a nickel-charged resin (Ni-NTA, Qiagen, Germany), and refolding was performed using a reverse urea gradient. The target protein was eluted from the column with buffer E (10 mM Tris-HCL pH7.5, 250 mM NaCl, 400 mM imidazole). After that, size-exclusion chromatography was performed on a Superdex 200 in GF buffer (20 mM HEPES pH 7.5, 250 mM NaCl) (Figure S2). The presence of the target protein was checked using SDS-PAGE (Figure S2).

#### 3.4.2. Expression and Purification of Recombinant VPg of Potato Virus Y

Cells of *E. coli* strain BL21(DE3)pRARE (Novagen, Germany) were transformed with plasmid pET22b(+)-VPg-PVY as in Section 3.4.1. Protein expression was induced by adding 0.3 mM IPTG (final concentration) when the culture OD590 reached ≈0.6–0.8. Cells were incubated for 20 h at +20 °C. The expression level was checked using SDS-PAGE.

To obtain PVY VPg, harvested cells were resuspended in buffer R1 supplemented with a cocktail of protease inhibitors (Thermo Fisher, USA), DNAse and PMSF. The cell suspension was disrupted by sonication in an ice-water bath. After disruption, the suspension was centrifuged at 10,000 rpm (13751 rcf) for 30 min at 4 °C. The target protein was present in the soluble fraction. This supernatant was applied to the column with a nickel-charged resin (Ni-NTA, Qiagen, Germany) and eluted from the column with gradient buffer E (0–100%). After that, size-exclusion chromatography was performed on a Superdex 75 in GF buffer (Figure S2). The presence of the target protein was checked using SDS-PAGE (Figure S2).

#### 3.4.3. Expression and Purification of Recombinant mCherry

Cells of *E. coli* strain BL21 Star (DE3) were transformed with the pET24-mCherry plasmid and grown on selective agar medium (50 µg/mL Kanamycin) for 16 h at +37 °C. A single colony from the plate was transferred into 10 mL of selective LB medium (50 µg/mL Kanamycin) and incubated overnight at +37 °C with shaking at 180 rpm. The overnight culture was subcultured into a preparative volume of selective medium (50 µg/mL Kanamycin) at a 1:50 ratio. Protein expression was induced by adding 0.3 mM IPTG (final concentration) when the culture OD590 reached ≈0.6–0.8. Cells were incubated for 3 h at +37 °C. Cells were harvested by centrifugation. Harvested cells were resuspended in buffer L (10 mM Tris-HCL pH 7.5, 400 mM NaCl) supplemented with DNAse, PMSF and protease inhibitors (Thermo Fisher, Massachusetts, WA, USA). The cell suspension was disrupted by sonication in an ice-water bath. After disruption, the suspension was centrifuged at 10,000 rpm (13751 rcf) for 30 min at 4 °C. The target proteins were present in the soluble fraction. This supernatant was applied to the column of a nickel-charged resin (Ni-NTA, Qiagen, Hilden, Germany). The target protein was eluted from the column with a gradient of buffer E (0–100%). After that, size-exclusion chromatography was performed on a Superdex 200 column in the same GF buffer as for PVY VPg purification. SDS-PAGE was performed to confirm the purity of the preparation.

#### 3.4.4. Expression and Purification of Recombinant mCherry-VPg

*E. coli* strain BL21 Star (DE3) was transformed with the plasmid pET24-mCherry-VPg and grown on selective agar medium (50 µg/mL Kanamycin) for 16 h at +37 °C. To obtain mCherry-VPg, we used the same protocol as for obtaining mCherry. Also the expression level was checked using SDS-PAGE.

Harvested cells were resuspended in buffer R1 (10 mM Tris-HCL pH 7.5, 250 mM NaCl, 10% glycerol) supplemented with DNAse, PMSF and protease inhibitors (Thermo Fisher, USA). The cell suspension was homogenized by sonication in an ice-water bath. After homogenization, the suspension was centrifuged at 10,000 rpm (13751 rcf) for 30 min at +4 °C. The target proteins were present in the insoluble fraction. Purification of mCherry-VPg was performed as in Section 3.4.1. After that, size-exclusion chromatography was performed on a Superdex 200 in GF buffer (Figure S2). The presence of the target protein was checked using SDS-PAGE (Figure S2).

### 3.5. SPR Analysis

Protein–protein interaction experiments were performed using surface plasmon resonance (SPR) on an imSPR-Pro system (iClueBio, Ansan-si, Gyeonggi-do, Korea) at the Core Shared Facility of the Institute of Protein Research, RAS. Only freshly purified proteins that had not been subjected to freeze–thaw cycles were used for all the measurements.

Recombinant proteins (mCherry-VPg or SleIF4E1-Δ1-54) were immobilized on the sensor COOH-Au chip (iClueBio, Korea). Four different concentrations of the analyte sample (SleIF4E1-Δ1-54 or PVY VPg, accordingly) were prepared by serial dilution in a running buffer containing 20 mM HEPES-NaOH pH 7.5, 250 mM NaCl, and 0.05% Tween-20. The analyte samples were injected at a flow rate of 30 μL/min. The injection step included a 300 s association phase followed by an 1800 s dissociation phase. BSA and recombinant mCherry were used as a negative control for non-specific protein binding. All the binding experiments were performed at 25 °C. Data were analyzed by globally fitting curves describing the simple 1:1 bimolecular model to the set of four sensorgrams using TraceDrawer software version 1.9.2 (BioPhysics, Ridgeview Instruments AB, Sweden, Uppsala). Each immobilization strategy and kinetic analysis was repeated at least three times.

## 4. Conclusions

Despite numerous studies on the eIF4E∙VPg interaction, the molecular mechanism underlying this protein–protein association remains poorly understood. Our experimental results provide unambiguous evidence that the α1–α2 hairpin of PVY VPg is involved in complex formation with eIF4E. We used a protein engineering approach to generate a chimeric protein. Unlike the conventional approach, which typically fuses two proteins via a linker, we integrated a fragment of one protein directly into the sequence of the other.

The chimeric mCherry-VPg construct binds to SleIF4E1-Δ1-54 with affinity comparable to that of full-length PVY VPg, demonstrating that the insertion does not disrupt the binding interface. This study thus provides a basis for mapping the VPg∙eIF4E interaction interface.

The chimeric construct greatly simplified and accelerated the measurement of affinity between eIF4E family proteins and PVY VPg. We therefore describe a universal platform that allows individual structural elements of proteins to be incorporated into a stable scaffold, facilitating expression and rapid kinetic characterization of protein–protein interactions.

## Figures and Tables

**Table 1:** Kinetic constants and equilibrium dissociation constants (K_D_) of the SleIF4E1-Δ1-54complexes with PVY VPg and mCherry-VPg. K_D_ = kd/ka.

№	Ligand	Analyte	ka (1/(M*s))	kd (1/s)	KD (nM)	SD (nM)
1	SleIF4E1-Δ1-54	PVY VPg	1.35 × 10^5^	4.45 × 10^−5^	0.33 ± 0.01	0.02
2	mCherry-VPg	SleIF4E1-Δ1-54	2.74 × 10^5^	6.95 × 10^−5^	0.25 ± 0.01	0.01
3	mCherry-VPg	BSA	ND	ND	ND	ND
4	mCherry	SleIF4E1-Δ1-54	ND	ND	ND	ND

ND—not detected. BSA—bovine serum albumin. KD values are presented as the mean of three independent experiments. The ± value represents the fitting error of the global analysis. The SD column reports the standard deviation calculated from the three independent replicate fits.

## Data Availability

The data presented in this study are available on request from the corresponding author.

## Supplementary Materials

The following supporting information can be downloaded at https://www.mdpi.com/article/10.3390/ijms27188187/s1.
