# Supplementary material for: Experimental Confirmation of the Role of the α1–α2 Hairpin PVY VPg in eIF4E Interaction
Source: Int J Mol Sci. 2026 Sep 15;27(18):8187. doi: 10.3390/ijms27188187 (PMC13607006; doi:10.3390/ijms27188187)
Supplement: Supplementary file 1 [file ijms-27-08187-s001.zip › figureS1.pdf]

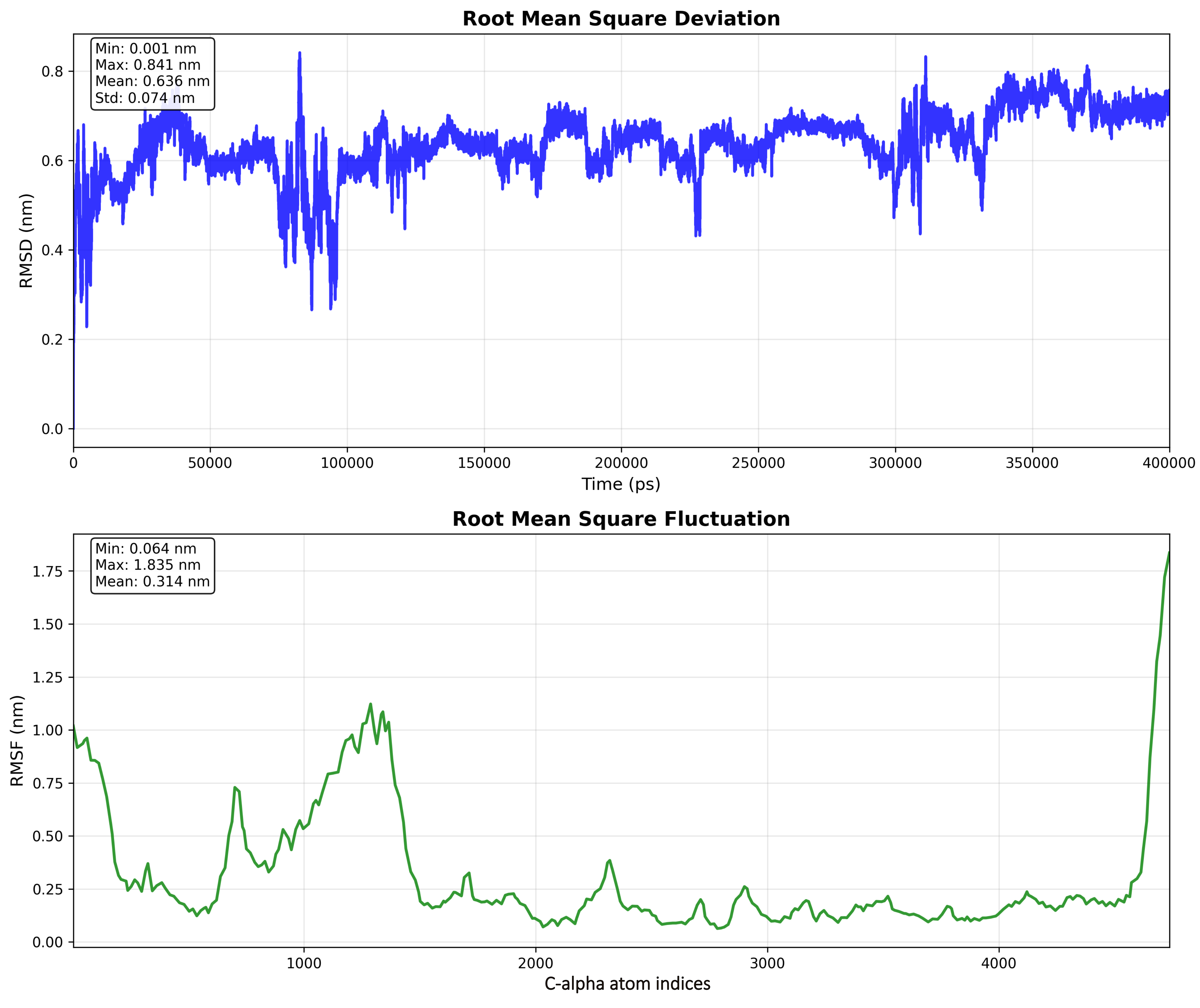

Figure S1. Assessment of conformational mobility. The top graph reflects the overall stability of the system (RMSD), while the bottom graph shows the local mobility of residues (RMSF calculated for C-alpha atoms)
