# Supplementary material for: Experimental Confirmation of the Role of the α1–α2 Hairpin PVY VPg in eIF4E Interaction
Source: Int J Mol Sci. 2026 Sep 15;27(18):8187. doi: 10.3390/ijms27188187 (PMC13607006; doi:10.3390/ijms27188187)
Supplement: Supplementary file 1 [file ijms-27-08187-s001.zip › FigureS2.pdf]

A SDS-PAGE analysis (left) of peak fractions from the SEC elution profile on a Superdex Increase 200 column (right), corresponding to SleIF4E1-△1-54

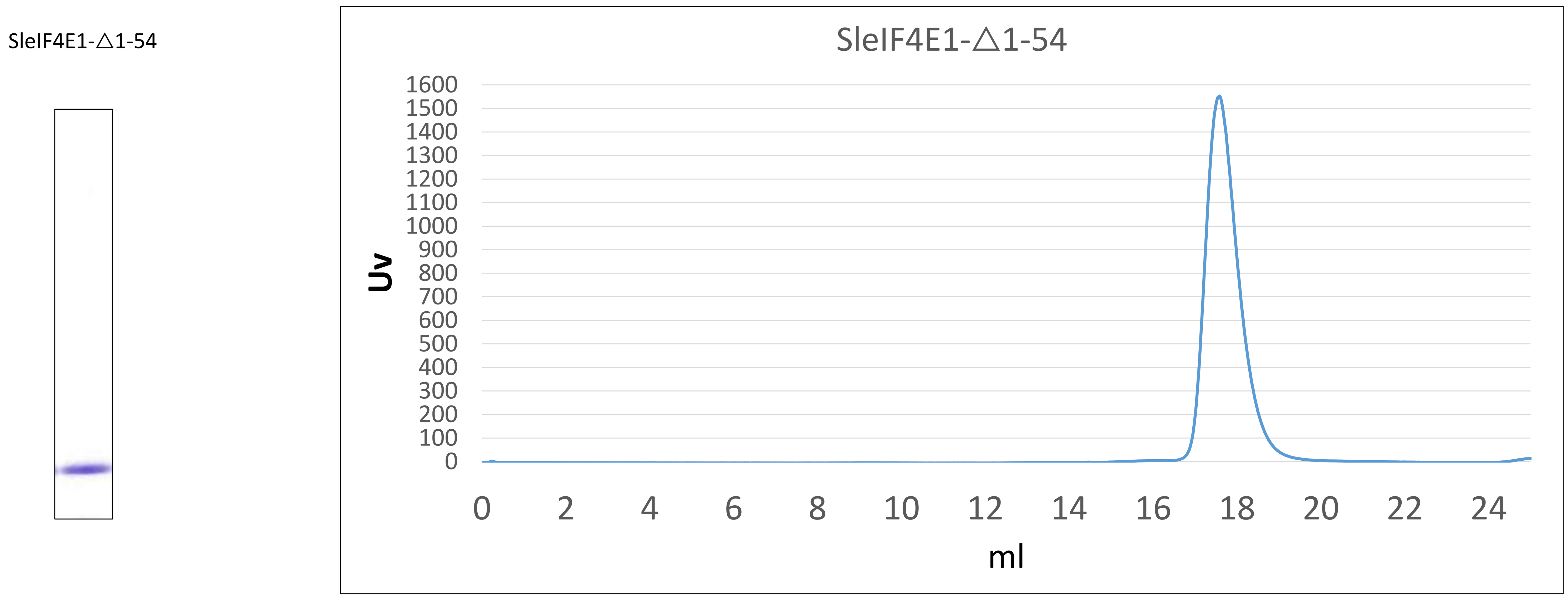

B SDS-PAGE analysis (left) of peak fractions from the SEC elution profile on a Superdex Increase 75 column (right), corresponding to PVY VPg

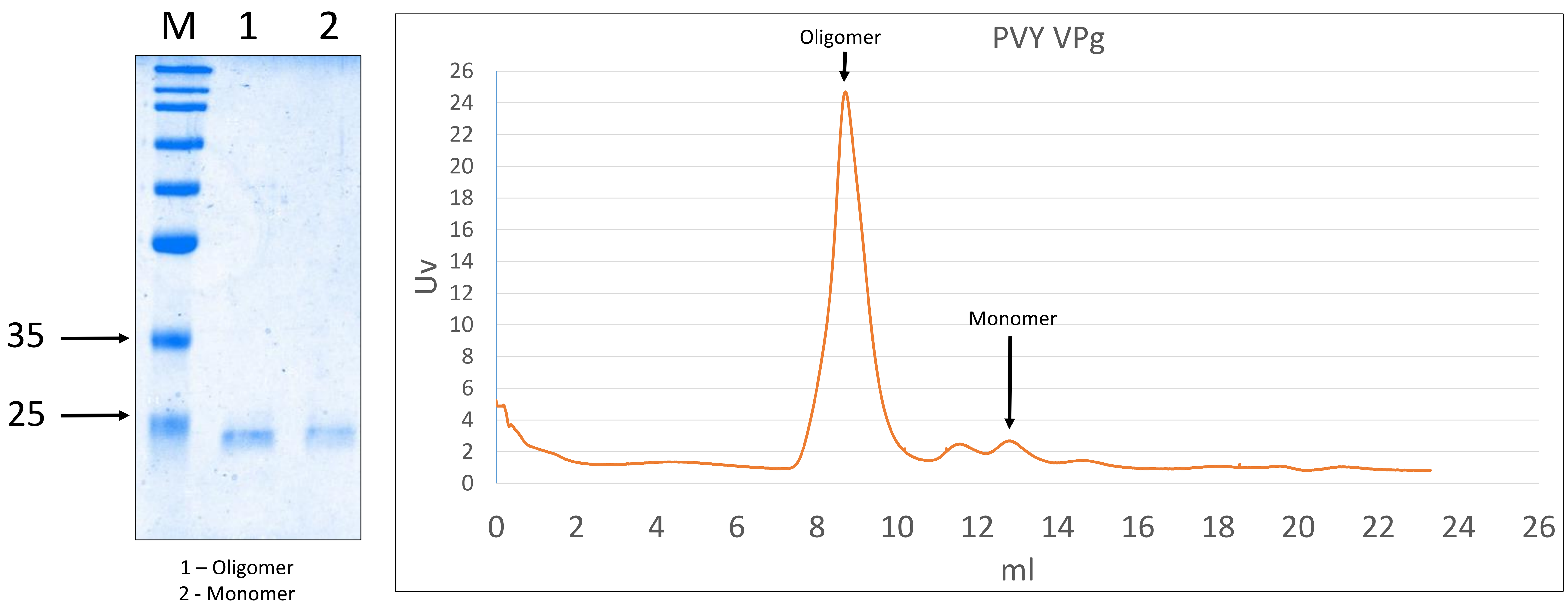

C SDS-PAGE analysis (left) of peak fractions from the SEC elution profile on a Superdex Increase 200 column (right), corresponding to mCherry-VPg

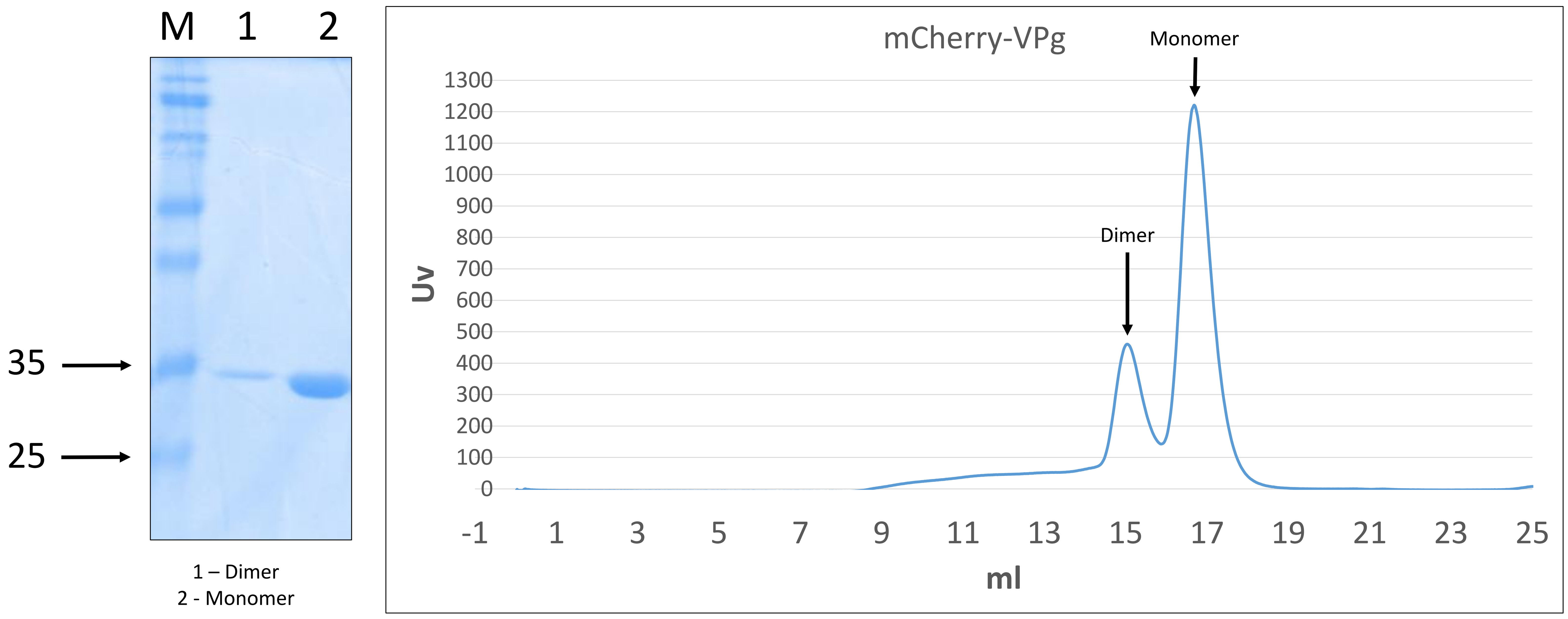

Figure S2: SDS-PAGE analysis of peak fractions from the SEC elution profile of the studied proteins

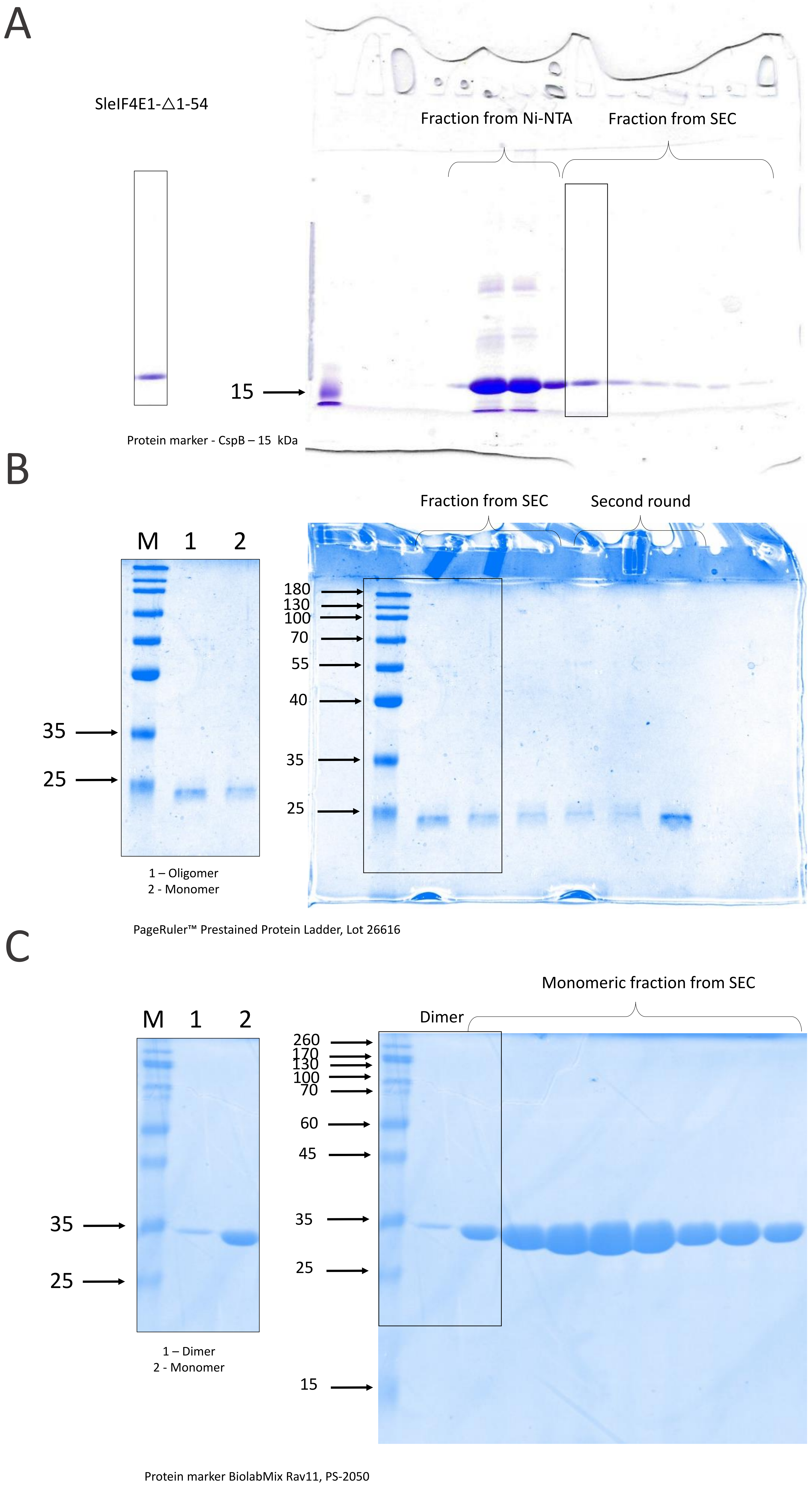
A
SleIF4E1-△1-54
Fraction from Ni-NTA
Fraction from SEC
15
Protein marker - CspB – 15 kDa
B
Fraction from SEC
Second round
M 1 2
180
130
100
70
55
40
35
25
35
25
1 – Oligomer
2 - Monomer
PageRuler™ Prestained Protein Ladder, Lot 26616
C
Monomeric fraction from SEC
M 1 2
Dimer
260
170
130
100
70
60
45
35
25
15
35
25
1 – Dimer
2 - Monomer
Protein marker BiolabMix Rav11, PS-2050
